# Fatty acyl-AMP ligases in bacterial natural product biosynthesis

**DOI:** 10.1039/d4np00073k

**Published:** 2025-02-19

**Authors:** Anne Liong, Pedro N. Leão

**Affiliations:** a CIIMAR – Interdisciplinary Centre of Marine and Environmental Research, University of Porto Matosinhos Portugal pleao@ciimar.up.pt; b ICBAS – School of Medicine and Biomedical Sciences, University of Porto Porto Portugal

## Abstract

Covering: covering up to 2024

Fatty Acyl-AMP Ligases (FAALs) belong to the family of adenylate-forming enzymes and activate fatty acyl substrates through adenylation. FAALs were discovered as key players in various natural product biosynthetic pathways, particularly in the assembly of polyketides and non-ribosomal peptides. These enzymes exhibit a conserved structural architecture that distinguishes them from their close relatives, the Fatty Acyl-CoA Ligases. FAALs display the starter unit in the biosynthesis of diverse natural products where they shuttle fatty acyl substrates into secondary metabolism for further chain elongation and/or modification. In this review, we cover the discovery, distribution and structure of FAALs as well as their role in natural product biosynthesis. In addition, we provide an overview about their genomic and biosynthetic contexts and summarize approaches used to analyze FAAL activity, predict their substrate specificity and to discover new compounds whose biosyntheses involve these enzymes.

## Abstract 2

FAAL enzymes play a crucial role in the biosynthesis of many natural products containing fatty acids. This review traces the history of FAALs, from their initial discovery to their widespread function in the biosynthesis of natural products.

## Graphical abstract

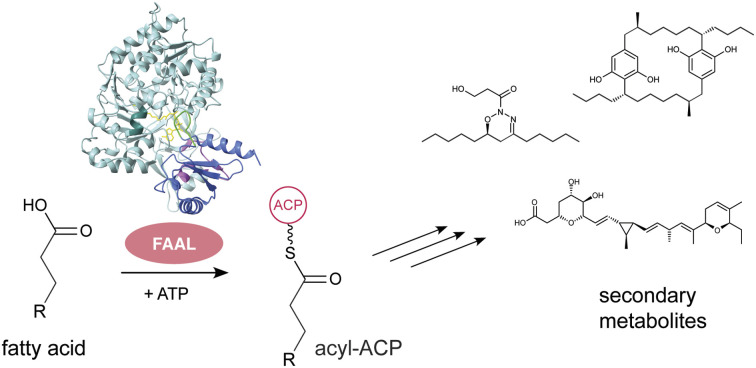

## 1 Introduction – adenylate-forming enzymes

**Fig. 1:**
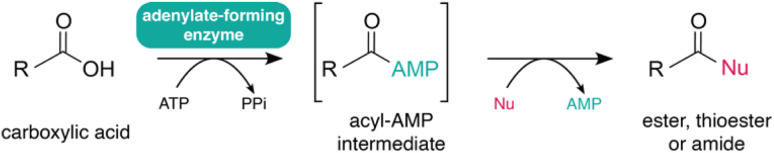
General reaction scheme for adenylate-forming enzymes. A carboxylic acid is activated by an adenylate-forming enzyme to form an acyl-AMP intermediate. After nucleophilic attack, an ester, thioester or amide is formed with the release of AMP.

In nature, fatty acids are usually activated before being assimilated into various metabolic pathways. Adenylation represents one of the most important reactions in biology, initiating the activation of fatty acids and other substrates in diverse metabolic pathways. The catalytic mechanism of the adenylation reaction was discovered over 60 years ago. Within this mechanism, carboxylate substrates are activated through condensation with adenosine triphosphate (ATP) setting free a highly reactive acyl adenylate (acyl-AMP) species. After reaction of a nucleophile (amide, alcohol or thiol), the final product is formed with release of AMP (Fig. 1).^1^

Across all three domains of life, adenylation is fulfilled by a superfamily of widely spread adenylate-forming enzymes (ANL). These are involved in diverse biochemical pathways such as protein synthesis, posttranslational modifications, amino acid- or fatty acid metabolism. A global analysis of protein family domains showed that ANL enzymes are the third most abundant domain in known natural products biosynthetic pathways.^2^ ANL enzymes can be divided into different classes based on their structural and catalytic properties.^3^ Class I includes acyl/aryl-CoA ligases (ACSs and ACLs), adenylation domains (A-domains) as part of non-ribosomal peptide synthetases (NRPSs), luciferases, aryl polyene adenylation enzymes,^4^ β-lactone synthetases^5^ and the most recently discovered group: fatty-acyl AMP ligases (FAALs).^6^ Class II comprises aminoacyl-tRNA synthetases. Class III consists of adenylating enzymes involved in siderophore synthesis.^7^ All three classes of enzymes are dependent on Mg^2+^ for their biological activity with the number of ions varying among them.^8,9^ Compared to class II (acting on amino acids) and class III (acting on dicarboxylic acid substrates), class I enzymes show promiscuity for a wider range of substrates such as fatty acids, amino acids, benzoic acids and their derivatives. The latter also do not show structural homology to class II or III enzymes.^3^ All members of class I (except luciferases) are characterized by the transfer of an adenylate to a 4′-phosphopantetheine (4′-Ppant) moiety, which happens through thioester bond formation with CoA or holo-acyl carrier protein (holo-ACP).^10^

**Fig. 2:**
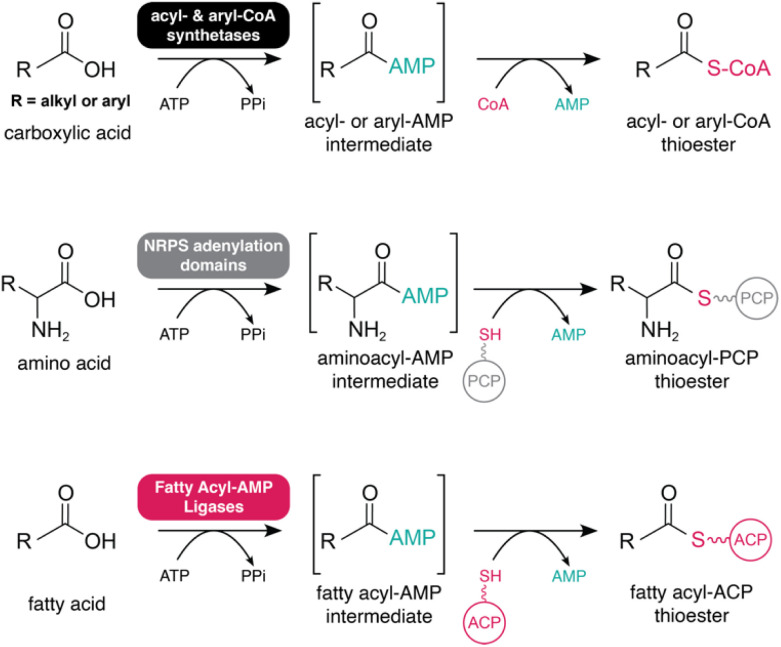
Reaction mechanism of ANL enzymes (acyl- and aryl-CoA synthetases, NRPS adenylation domains and fatty acyl-AMP ligases). All reactions include the formation of an acyl-AMP intermediate, followed by nucleophilic attack and formation of a thioester with release of AMP.

In this review, we focus on FAALs, enzymes (or domains) found in secondary metabolite biosynthetic gene clusters (BGCs), as an atypical enzyme system within the ANL superfamily. FAALs transfer activated fatty acyl substrates to a holo-ACP (Fig. 2), and prime the biosynthesis for a diversity of natural product families, including lipopeptides, different polyketide classes, as well as other lipids. We cover current knowledge about FAAL distribution among the tree of life, following their discovery in *Mycobacterium tuberculosis* (Mtb) and summarize recent research on FAAL structure and how this relates to their unique function. We then describe examples of bacterial biosynthetic pathways that involve FAALs, of the breadth of structures that can be generated in this way, and also of how FAAL activity can be ascertained. Finally, we showcase methods that have and can be used to uncover metabolites produced by FAAL-containing pathways.

## 2 Discovery, distribution, and structure of FAALs

### 2.1 Discovery of fatty acyl-AMP ligases

**Fig. 3:**
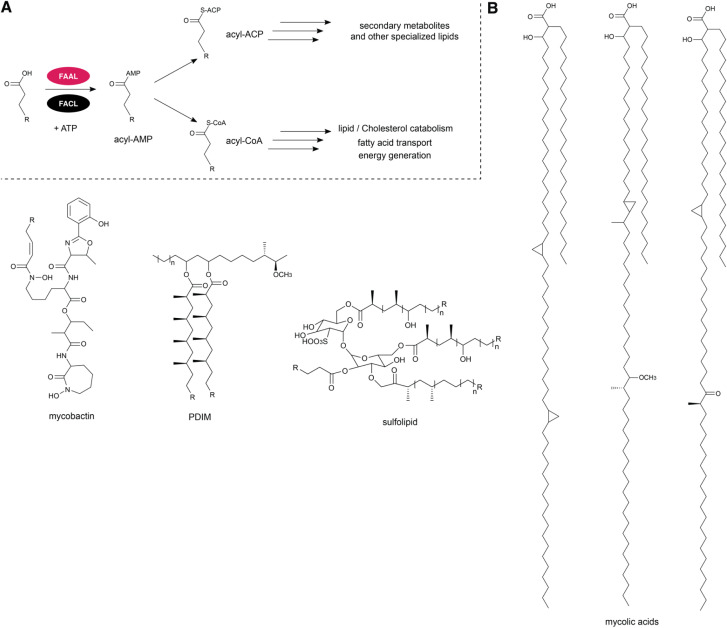
FAALs and FACLs function in Mtb. (A) FAALs use fatty acids to generate mycobacterial secondary metabolites while FACLs use them to generate fatty acyl-CoA followed by utilization in lipid- and cholesterol catabolism, for fatty acid transport and energy generation. (B) Structures of mycobacterial metabolites that involve FAALs in their biosynthesis: mycobactin, PDIM, sulfolipid and mycolic acids.

FAALs were characterized for the first time in Mtb by Arora and colleagues^6^ through bioinformatics approaches that included analysis of the Mtb genome. In Mtb, Fatty Acyl-CoA Ligases (FACLs) participate in lipid and cholesterol catabolism, fatty acid transport and energy generation while FAALs are involved in the biosynthesis of various essential complex virulence-conferring lipids such as sulfolipids, mycobactin and mycolic acids (Fig. 3A). Those metabolites are essential for the survival and virulence of the bacterium under harsh environmental conditions.^11^ For example, in the cell envelope of mycobacteria, the biosynthesis of mycolic acids is dependent on the presence of FAALs linking the incorporation of fatty acids with polyketide synthesis.

Mycolic acids are produced by a two-step catalytic reaction that begins with the activation of fatty acid carboxyl residues by adenosine triphosphate (ATP) hydrolysis, followed by a transfer to the acceptor molecule holo-ACP.^12^ In Mtb, fatty acids are transferred by a group of FAALs onto ACP domains belonging to type I polyketide synthases (PKSs) to produce lipidic metabolites.^13^ For example, FAAL32 (also known as FadD32) is required for the activation of a meromycolic acid chain in mycolic acid biosynthesis and thus essential for mycobacterial growth.^14^ Other FAALs such as FAAL26, FAAL28 and FAAL29 are involved in the biosynthesis of complex polyketides and glycolipids such as phthiocerol dimycocerosates (PDIMs) and phenolic glycolipids (PGLs). PDIMs are major virulence lipids in the cell wall of Mtb and are involved in the immune evasion strategies of Mtb helping the pathogen with survival within host macrophages.^15^ More precisely, the biosynthesis of PDIM is encoded by a 44 kb cluster that consists of five modular PKSs where FadD26 activates long chain fatty acids and transfers them onto the first ACP domain of the adjacent PKS enzyme PpsA. Thus, biosynthesis of the phthiocerol moiety is fulfilled by crosstalk between a FAAL (FadD26) and the *pps* modular PKS cluster.^16,17^ To generate PDIM, mycocerosic acid moieties are installed by FAAL28. Interestingly, the PGL cluster in Mtb contains two FAALs, FAAL29 and FAAL22. While FAAL29 shows overlapping substrate profile for long chain fatty acids, FAAL22 has unusual specificity for *p*-hydroxybenzoic acid. The biosynthesis of phthiocerol or the phenolphthiocerol chains for DIMS and PGLs includes the loading of *p*-hydroxybenzoic acid and synthesis of *p*-hydroxyphenylalkanoic acid that is used as a substrate for FAAL29 and loaded onto PpsA-E for elongation.^6,18,19^ Besides FAAL22 and FAAL29 that assemble the phenolphthiocerol lipid part, FAAL28 is needed for the installation of the two mycocerosic acid units to form PGLs (Fig. 3B).^20^

Hence, the discovery and characterization of FAALs in Mtb significantly advanced the understanding of complex lipid biosynthesis pathways crucial for the virulence of this human pathogen and underlines FAALs as main target for antitubercular drug development. Concomitantly, it set the stage for understanding the diversity of these enzymes in other organisms.

### 2.2 Distribution of FAALs in bacteria and eukaryotes

FAALs are present in both bacteria and eukaryotes, where they play essential roles in lipid metabolism.^21^ In bacteria, FAAL members are not only encoded near PKS I gene clusters as stand-alone proteins but also at the N-terminus of NRPSs.^22^ These two classes of large multimodular enzymes can together catalyze condensation reactions of more than 500 different monomers including β-keto acids, proteinogenic and non-proteinogenic amino acids, fatty acids and α-hydroxy acids leading to a variety of diverse bioactive molecules.^23^ Genome predictions on the genome of Mtb led to the identification of over 18 FAAL domains, several of them with so-far unexplored functions.^24^ FAAL-encoding genes have been identified in bacteria, notably in actinobacteria, cyanobacteria and proteobacteria as well as in eukaryotes, most being genomically located near ACPs.^25^

Looking at the distribution in bacteria, FAALs have been most extensively studied in Mtb but are also present in other members of the actinobacteria phylum. Homologues could be identified in mycolic acid-producing actinobacteria such as *Nocardia farcinica* and *Rhodococcus* sp. RHA1,^6^ as well as in *Corynebacterium glutamicum*,^26^ but also lipopeptide-producing bacteria such as *Bacillus subtilis* and several cyanobacteria encode FAALs or proteins with FAAL domains.^27,28^

When compared to bacteria, FAALs are relatively rare in eukaryotes. Still, a set of FAAL sequences could be identified in eukaryotes such as fungi, algae, plants, stramenopiles and alveolates.^25^ Interestingly, in the genome of *Homo sapiens*, proteins named disco-interacting protein 2 homologue C (DIP2C) harbouring two FAAL-like domains have been identified. Still, these protein domains showed only 25.0% and 26.75% of sequence identity with well characterized FAALs from *Legionella pneumophila*.^29^ FAAL sequences have also been identified in metagenome assembled genomes (MAGs) obtained from coastal permeable sediment samples – FAALs were present in the MAGs of the archaeal taxa of *Halobacteria archaeon* (ncbi accession numbers NNJ93261.1 and NNJ93262.1).^21^ The presence of FAALs in archaea might be interesting out of several reasons such as evolutionary significance, metabolic diversity and adaptability of these microorganisms. Archaea often inhabit extreme environments such as those with high temperatures, high salt concentrations or acidic conditions and this has been linked to their differentiated lipid profile, when compared to bacterial or eukaryotic lipids (*e.g.* ether-linked *vs.* ester-linked lipids).^30^ The presence of FAAL in all kingdoms of life may suggest an early evolution and presence in LUCA (Last Universal Common Ancestor). The existing literature does not investigate further how FAAL genes evolved.

In summary, FAALs are primarily found in bacteria, especially within the phylum actinobacteria. Although their presence in eukaryotes and archaea is more restricted, the FAAL domain seems to be utilized across the tree of life in lipid metabolism.

### 2.3 Structural analysis of FAALs compared to FACLs

Before the discovery of key differences in amino acid sequence and structure, several FAALs have been wrongly annotated as FACLs. For example, this was the case for FadD10, which is involved in the biosynthesis of a virulence-related lipopeptide in Mtb.^31^ Among 34 FadD proteins identified in the Mtb genome, bioinformatics analysis followed by biochemical characterization suggested that 12 of these proteins could be classified into the group of FAALs.^12^ Analysis of the 3D-structures of the enzymes proved useful for classification. The first insights into FAALs 3D-structure were obtained by solving a crystal structure of the N-terminal domain of FAAL28 from Mtb. Due to difficulties in crystallization, only the N-terminal structure of the protein could be obtained *via* X-ray crystallography.^6^ The first full-sequence crystal structures of FAALs were obtained from *Escherichia coli*, EcFAAL and *L. pneumophila*, LpFAAL, and included the acyl adenylates bound in their active sites (lauroyl-adenylate and myristoyl-adenylate in EcFAAL; lauroyl-adenylate in LpFAAL).^32^ Ever since, various 3D-structures of FAALs from different bacteria were made available to the public (Table 1).

**Table 1:** List of FAALs that have been structurally characterized *via* X-ray crystallography

Protein	Organism	PDB-ID	Substrate	Reference
FadD28 N-terminal	*M. tuberculosis*	3E53	Long-chain fatty acids	6
FadD28 N-terminal G330W	*M. tuberculosis*	3T5A	Long-chain fatty acids	33
FadD13 N-terminal	*M. tuberculosis*	3T5B, 3T5C, 5ZRN	Long-chain fatty acids	33
FadD23 N-terminal	*M. tuberculosis*	8HCZ	Mainly C16:0	34
FadD10	*M. tuberculosis*	4ISB	Mainly C14:0	31
FadD23	*M. tuberculosis*	8IQU	Mainly C16:0	34
FadD32	*M. tuberculosis*	5HM3	Mainly C14:0	35
EcFAAL	*E. coli*	3PBK	C12:0, C14:0	32
LpFAAL	*L. pneumophila*	3KXW, 3LNV	C12:0	32
FAAL domain R336A of PKS	*Picosynechococcus* sp. PCC 7002	7R7F	Unknown	To be published
FAAL domain A229/R336A of PKS	*Picosynechococcus* sp. PCC 7002	7R7G	Unknown	To be published
FAAL domain of PKS	*Picosynechococcus* sp. PCC 7002	7R7E	Unknown	To be published
FadD32	*Mycobacterium marinum*	5EY9	C12:0, C14:0	36
FadD32	*Mycobacterium smegmatis*	5EY8	C12:0, C14:0	36
FAAL	*Mycobacterium smegmatis*	5D6N	Unknown	37

Overall, FAALs share a common domain organization, namely an N-terminal domain that contains an adenylation domain and a C-terminal domain. The N-terminal domain is responsible for binding and recognizing fatty acid substrates and catalyzes the acyl transfer to the ACP. More precisely, the adenylation domain catalyzes the conversion of fatty acids into acyl-AMPs by binding ATP and facilitating the formation of the acyl-AMP intermediate while the C-terminal domain is involved in enzyme stabilization and interacts with the ACP to mediate docking and alignment for the acyl-transfer.^12,38^

**Fig. 4:**
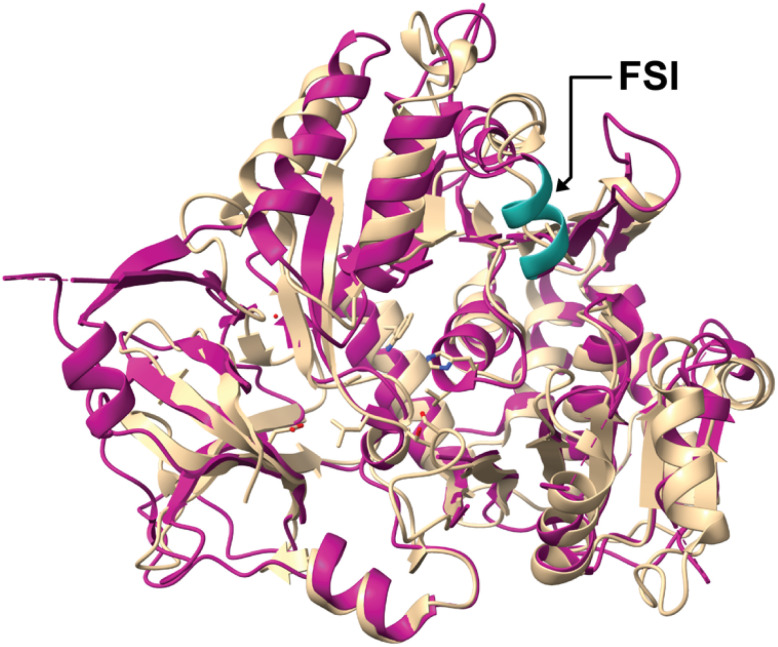
Structural alignment of N-terminal domain of *M. tuberculosis* FAAL28 (magenta, PDB-ID: 3E53) with N-terminal domain of FACL13 (beige, PDB-ID: 5ZRN) highlighting the FAAL specific insertion sequence (FSI, turquoise).

FAALs share structural features with FACLs that ligate fatty acids to Coenzyme A (CoA) *via* a thioester bond while FAALs load fatty acids onto their cognate ACP. Surprisingly, studies showed the preference of FAALs for holo-ACP but not for CoA although both contain the protruding Ppant group. It was proposed that the FAAL specific insertion (FSI), a stretch of several amino acids in the N-terminus of the FAALs but not in the FACLs, might be preventing domain rotations resulting in the formation of acyl-CoA derivatives (Fig. 4).^6^ Studies demonstrated that the introduction of the FSI from Mtb into a FACL results in decreased acyl-CoA production. To confirm these results and to understand if acyl-CoA formation is dependent on the FSI, Arora *et al.* and Goyal *et al.*^6,33^ tested the deletion of the sequence in Mtb-FAAL which resulted in a weak acyl-AMP reaction with CoA, leaving the inner workings of this mechanism still not fully understood.

In another recent study it was found that FAALs use highly selective alternative binding sites that can distinguish 3′,5′-bisphosphate-containing CoA from holo-ACP, thus ensuring CoA-independent activation and redirection of fatty acids towards the biosynthesis of lipidic metabolites. Still, the exact mechanism leading to the observed selectivity is not yet fully understood.^25^ The presence of FAAL-like proteins in bacteria, archaea and eukaryote genomes led to the conclusion that formation of acyl-ACPs by these enzymes is relevant in lipid metabolite biosynthesis across the tree of life, and was evolutionary mediated by the addition of a FAAL specific insertion sequence into FACLs.^33^

In both FAALs and FACLs, the larger N-terminal domain is connected to the smaller C-terminal domain through a flexible loop. This flexibility enables the C-terminal domain to adopt different orientations, facilitating substrate binding and, in FAALs, interactions with the ACP or, in FACLs, with CoA for fatty acyl group transfer. Additionally, the N-terminal domain is divided into three subdomains that form a pocket for substrate and product binding.^32^ Structural features of ANL family members were compared by Patil *et al.*^25^ and the study showed that FAALs interact with their ligands through (i) hydrogen bonds formed by residues of the C-terminal protein domain, (ii) water-mediated contacts formed by N-terminal helices and (iii) interactions with phosphates through positively charged residues (Arg/Lys). Structure-alignments of different FAALs, namely FadD32 from *Mycobacterium marinum*, FadD32 from *Mycobacterium smegmatis*, FadD28, FadD13 and FadD10 from Mtb, FAALs from *L. pneumophila* and *E. coli*, were generated by Guillet *et al.*^36^ and compared with FACLs. All analyzed FAALs contained a conserved Ile-363-Val-386 SI4 segment that builds the bridge between N- and C-terminal domains.^32^ In previous studies, it was shown that this motif is a specific trait of FAAL homologs, sufficient to prevent the formation of acyl-CoA derivatives.^6^

**Fig. 5:**
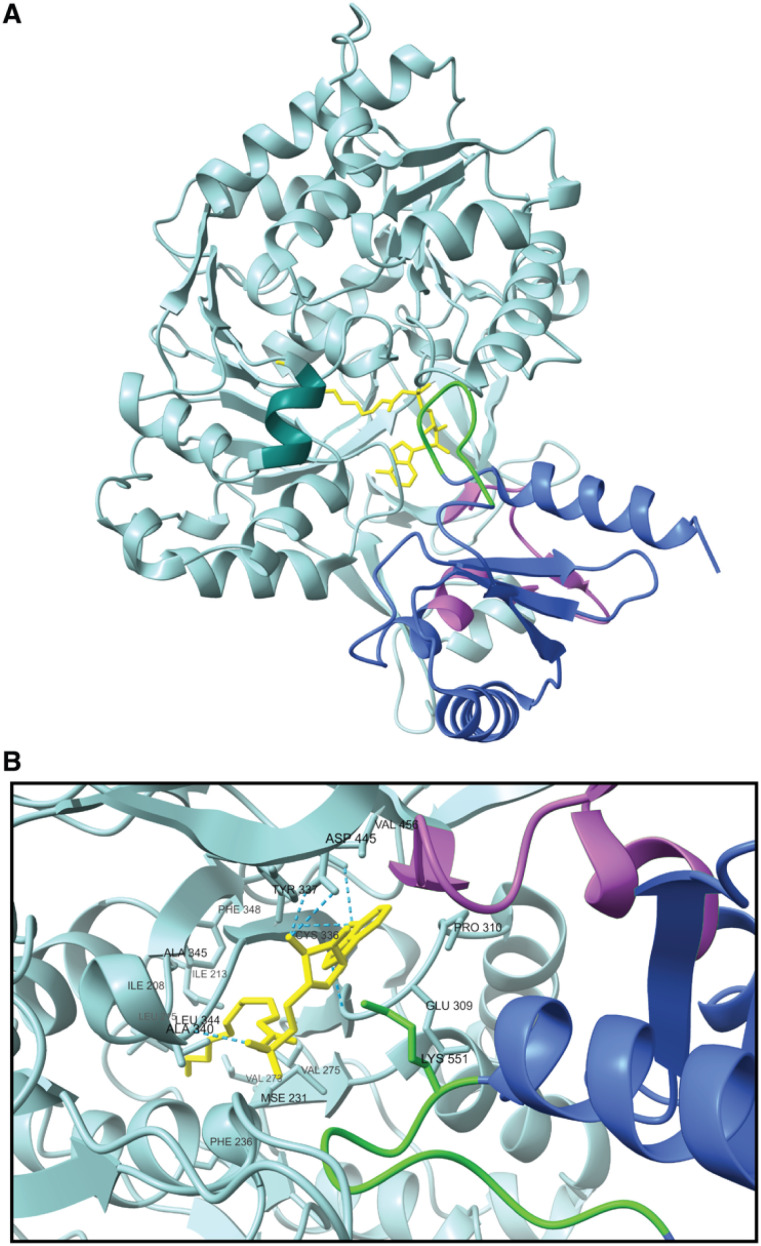
(A) Structure of chain A of EcFAAL (PDB-ID: 3PBK), ligand 5′-*O*-[(*S*)-(dodecanoyloxy)(hydroxy)phosphoryl]adenosine in yellow, N-terminal domain in light blue, hinge region in pink, C-terminal domain in blue, highly conserved loop region reaching into the active site in green and FSI in turquoise. (B) Zoom in into active site of EcFAAL.

Because crystallization of EcFAAL and LpFAAL led to co-crystallization of the acyl adenylate bound to the enzyme, 3D-structures could be analyzed regarding substrate and product binding (Fig. 5A). According to structural analysis, three loops form the entrance to the active site, while residues 545–553 (annotation according to EcFAAL) from the C-terminal domain may belong to the channel reaching the active site of the enzyme. During substrate binding and product dissociation, the highly flexible hinge region enables C-terminal domain movements. In both analyzed structures, the AMP moieties seem to interact with conserved residues, while the adenyl rings are bound in a hydrophobic pocket supported by hydrogen bonds formed with main and side chain atoms (Fig. 5B).^32^

The hydrophobic pocket in EcFAAL is primarily formed by Pro310, Tyr337 and Val456. Further substrate positioning is achieved through hydrogen bonds between the adenyl ring and Cys336 in the N-terminal domain as well as hydrogen bonding of the ribose moiety to Asp445. Extensive van der Waals interactions also play a crucial role in stabilizing the substrate within the enzyme's pocket. Additionally, the phosphate oxygens are bound to Ala340 *via* hydrogen bonds, supporting the overall stability of the substrate binding. These interactions are conserved among FAAL enzymes and ensure efficient substrate binding and positioning.^32^

## 3 Role of FAALs in natural products biosynthesis

### 3.1 Genomic context of FAALs and their ACPs in bacteria

In bacteria, FAAL-encoding genes are often found within BGCs associated with the multimodular PKS and NRPS machineries. These encode for the biosynthesis of complex lipidic and peptidic natural products, respectively, and are often found in both Gram-positive and Gram-negative bacteria.^12^ FAALs play a key role in initiating biosynthesis by activating fatty acids and loading them onto ACPs; the resulting thioester is then primed for nucleophilic attack by the activated monomer in the ensuing module. In most cases, the FAAL-encoding gene is located near genes coding for ACPs or is directly associated with an ACP-domain in a didomain protein. This ensures the efficient transfer of activated fatty acid to the rest of the biosynthetic machinery. But FAAL domains can be also part of multidomain proteins as recently described for the biosynthesis of occidiofungins, antifungal natural products of hybrid PKS III/NRPS origin in *Burkholderia* species.^39^ In Cyanobacteria and Pseudomonadota, FAALs fused to either PKS (*e.g.* in desmamides,^40^ micacocidin^41^) or NRPS (*e.g.* vioprolides^42^) were also described. A more detailed discussion on this topic is provided in below, together with illustrative examples.

To date, a large variety of FAAL-encoding gene clusters have been identified and this number is increasing, as advances in bioinformatics lead to better annotation of FAAL domains.^43^ Taking into account both published fatty acyl-chain containing natural products and the MIBiG database,^44^ most of the so far characterized fatty acyl-containing natural products associated with FAAL-containing pathways originate from the phyla Cyanobacteria, Pseudomonadota, Actinobacteria, Firmicutes, Myxococcota and Bacteroidetes (Table 2).

**Table 2:** Examples of published natural products associated with FAAL-encoding biosynthetic gene clusters. Each biosynthetic gene cluster encodes at least one FAAL enzyme/domain

Compound name	Substrate	Evidence level	Phylum	Reference
Cylindrocyclophanes	C10:0	*In vitro* data	Cyanobacteria	45
Hectochlorin	C6:0	Hypothesis	Cyanobacteria	46
Jamaicamides	C6:1, C6:2	*In vitro* data	Cyanobacteria	47
Malyngamides	C8:0	Hypothesis	Cyanobacteria	48
Nocuolin A	C6:0, C8:0	*In vitro* data	Cyanobacteria	28
Hapalosin	C8:0	Hypothesis	Cyanobacteria	49
Puwainaphycin	C12:0–C16:0	Hypothesis	Cyanobacteria	50
Microginins	C10:0	Hypothesis	Cyanobacteria	51
Columbamides	C12:0, C14:0	Hypothesis	Cyanobacteria	52
Desmamides	C6:0	Hypothesis	Cyanobacteria	40
Olefin	Variable	Hypothesis	Cyanobacteria	53
Hassalidins	C14:0, C16:0	Hypothesis	Cyanobacteria	54
Minutissamides	C12:0–C16:0	Hypothesis	Cyanobacteria	55
Laxaphycin	C6:0	Hypothesis	Cyanobacteria	56
Chlorinated lactylates	C12:0	Hypothesis	Cyanobacteria	57
Scytocyclamides	C6:0	Hypothesis	Cyanobacteria	58
Vatiamides	C6:0	Hypothesis	Cyanobacteria	59
Carbamidocyclophanes	C10:0	Hypothesis	Cyanobacteria	60
Merocyclophane C, D	C10:0	Hypothesis	Cyanobacteria	61
Chlorosphaerolactylates A–D	C12:0	Hypothesis	Cyanobacteria	57
Nocuolactylates	C12:0	*In vitro* data	Cyanobacteria	28
Fischerazoles A–C	C16:0	Hypothesis	Cyanobacteria	62
Nostovalerolactones	C8:0	Hypothesis	Cyanobacteria	63
Ralsolamycin	C16:0	Hypothesis	Pseudomonadota	64
Micacocidin	C6:0	*In vitro* data	Pseudomonadota	65
Ambruticin	C14:0, C16:0	*In vitro* data	Pseudomonadota	66
Tambjamine	C12:0	*In vitro* data	Pseudomonadota	67
Alkylresorcylic acid	C18:0	*In vitro* data	Pseudomonadota	68
Caryoynencin	C18:1	Hypothesis	Pseudomonadota	69
Vioprolide	C16:0, C17:0	*In vitro* data	Pseudomonadota	42
Gramibactin	C8:0	Hypothesis	Pseudomonadota	70
Trinickiabactin	C8:0	Hypothesis	Pseudomonadota	71
Histicorrugatin	C8:0	Hypothesis	Pseudomonadota	72
Taiwachelins	C12:0	Hypothesis	Pseudomonadota	73
Fabrubactin A, B	C10:0	*In vitro* data	Pseudomonadota	74
Variochelin A, B	C12:0	Hypothesis	Pseudomonadota	75
Plantaribactin	C12:0	Hypothesis	Pseudomonadota	76
Ralsolamycin	C16:0	Hypothesis	Pseudomonadota	77
Occidiofungins	C18:0	Hypothesis	Pseudomonadota	39
Serobactin	C10:0–C14:0	Hypothesis	Pseudomonadota	78
Rakicidin D	C6:0	Hypothesis	Pseudomonadota	79
Massiliachelin	C6:0	Hypothesis	Pseudomonadota	80
Mycolic acid	C14:0	*In vitro* data	Actinobacteria	81
PDIM	C29:0, C30:0, C32:0 (branched)	Hypothesis	Actinobacteria	16
Glycopeptidolipid	C26:0–C34:0	Hypothesis	Actinobacteria	82
Daptomycin	C8:0–C14:0	*In vitro* data	Actinobacteria	83
Taromycin	C8:0	Hypothesis	Actinobacteria	84
Catenulisporolides	C5:0	Hypothesis	Actinobacteria	85
Telomycin	C8:0	Hypothesis	Actinobacteria	86
Rotihibin A	C10:0	Hypothesis	Actinobacteria	87
Sulfolipids	C24:0–C34:0	Hypothesis	Actinobacteria	6
Naphthyridinomycin	C14:0	*In vitro* data	Actinobacteria	88
Mycosubtilin	C16:0	Hypothesis	Firmicutes	89
Mycobactin	C14:0–C18:0	Hypothesis	Firmicutes	90
Bacillomycin D	C14–C17 β-amino fatty acid	Hypothesis	Firmicutes	91
Iturin A	C14:0–C17:0	Hypothesis	Firmicutes	92
Chondrochloren A	C4:0	Hypothesis	Myxococcota	93
Ambruticins	C14:0, C16:0	*In vitro* data	Myxococcota	66
Alkylpyrone-407	C18:0	*In vitro* data	Myxococcota	94
Alkylpyrone-393				
Corramycin	C6:0–C12:0	*In vitro* data	Bacteroidetes	95

### 3.2 Stand-alone FAALs

**Fig. 6:**
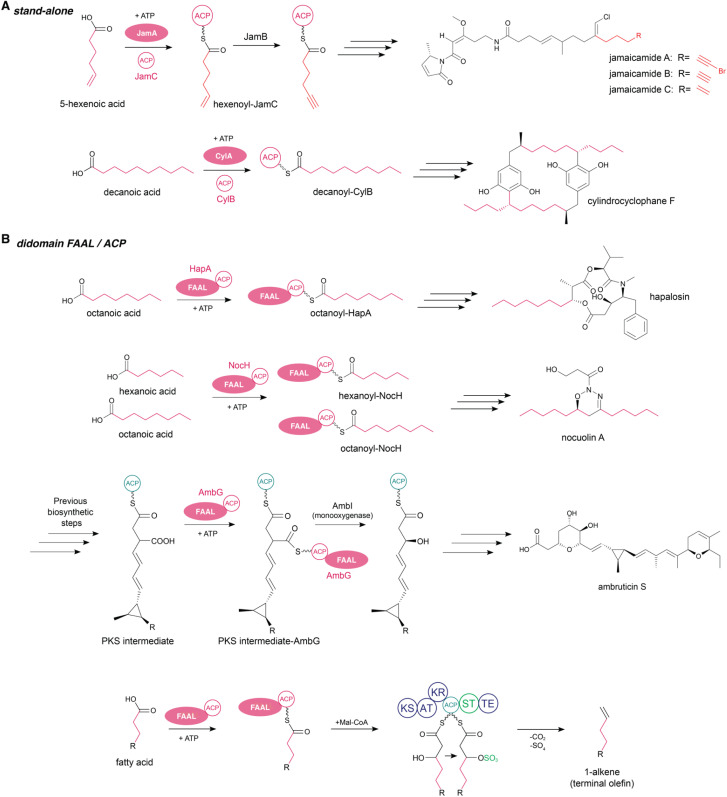
Examples of FAALs as stand-alone, didomain and part of multidomain enzymes. (A) Stand-alone FAALs JamA in jamaicamides and CylA in cylindrocylophanes biosynthesis, (B) didomain FAALs HapA in hapalosin, NocH in nocuolin A and AmbG in ambruticin S biosynthesis, KS: ketosynthase, AT: acyltransferase, KR: ketoreductase, ST: sulfotransferase, TE: thioesterase.

FAAL-encoding genes are often stand-alone, despite usually being co-localized with their cognate ACP. This is the case in jamaicamide biosynthesis, which we use here as an example (Fig. 6A). Jamaicamides are a group of highly functionalized lipopeptides from cyanobacteria that contain alkynyl bromide, vinyl chloride, beta-methoxy enone, and pyrrolinone ring functionalities. The fatty acid-incorporating enzyme of this BGC is a stand-alone FAAL, JamA, which was initially annotated as an acyl-ACP synthetase.^47^ In the biosynthesis of these compounds, a 5-hexenoic acid unit is activated by JamA and loaded onto the ACP JamB as an acyl-ACP thioester. A desaturase then acts on the alkyne moiety to generate a 5-hexynoyl-ACP and this unit is then further elongated and modified through PKS and NRPS machinery as well as tailoring enzymes.^47,96^ Jamaicamide biosynthesis illustrates the richness of enzymatic transformations associated with fatty acid moieties in secondary metabolism.^97^ The cylindrocyclophanes are another example of a natural product in which a stand-alone FAAL initiates biosynthesis, in this case interfacing with type III PKS machinery (Fig. 6A). Here, the FAAL loads decanoic acid onto its ACP which is then elongated by type I PKS machinery followed by elongation and cyclization by the encoded type III PKS enzyme.^45,98^

### 3.3 Didomain FAALs

FAAL domains are also found fused with their cognate ACP in a didomain protein (Fig. 6B). A canonical example for such a didomain arrangement can be found in the hapalosin BGC where FAAL initiates the biosynthesis through loading of an octanoic acid onto its ACP followed by transfer to a type I PKS and further elongations and modifications by several type I PKS and NRPS modules.^49^ A non-canonical example is the nocuolin A BGC, which also encodes for a fused FAAL-ACP protein (NocH). In the biosynthesis of nocuolin A two fatty acyl-NocH thioesters are simultaneously used by the downstream ketosynthase NocG to form a beta-keto acid featuring a 13-carbon chain. So far it is the only known biosynthesis where the same FAAL-ACP is used twice, loading two different substrates, namely hexanoic- and octanoic acid.^99^

Another example for the presence of a non-canonical FAAL-ACP didomain is AmbG in ambruticin biosynthesis.^66^ AmbG accepted a diverse range of substrates ranging from fatty acids to structurally diverse carboxylic acids including functionalized fatty acids and unsaturated and aromatic carboxylic acids. AmbG is the first example of a FAAL-ACP didomain that is centrally located in a type I PKS and able to activate polyketidic intermediates.^66^

Didomain FAAL-ACP enzymes in association with PKS enzymes are also involved in hydrocarbon and lipid metabolism, namely in the extensively studied terminal olefin biosynthesis. The olefin synthase (OLS) pathway is present in several different clades of cyanobacteria and usually consists of a FAAL and ACP, followed by a modular ketosynthase (KS), acyltransferase (AT), ketoreductase (KR) and ACP domains followed by a separate sulfotransferase (ST) and thioesterase (TE) module. This BGC architecture is like the FAAL-PKS architecture in Mtb. After loading of a fatty acid onto the ACP, the KS domain extends the fatty acyl-ACP *via* an acetate unit and reduces the beta-carbonyl to a hydroxy group, followed by decarboxylation and desulfation to create a terminal double bond resulting in the biosynthesis of odd-chain length hydrocarbons with a terminal olefin.^100^ However, in some cyanobacterial strains such as *Cyanobacterium stanieri* PCC 7202, the FAAL involved in olefin biosynthesis is a stand-alone protein.^53^

### 3.4 FAALs as part of multidomain enzymes

**Fig. 7:**
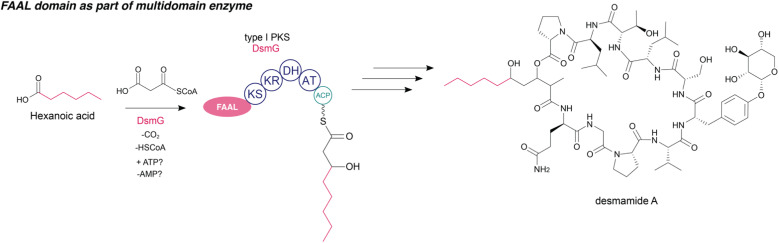
FAAL as part of multidomain enzymes in desmamide A biosynthesis. KS: ketosynthase, KR: ketoreductase, DH: dehydrogenase, AT: acyltransferase.

Because FAALs eventually form acyl thioesters, these can recruit and load FA starter units to initiate biosynthesis in PKS or NRPS assembly lines, instead of the more typical initiation through acyltransferase (AT) or adenylation (A) domains, respectively.^101^ As mentioned above, A domains (which usually activate and load amino acids) and FAALs are members of the same protein superfamily but they only share moderate sequence homology. Besides conserved motifs responsible for ATP-binding, divergence occurs in their substrate-binding regions.^3^ Nevertheless, such embedded FAAL domains can be misannotated by bioinformatics pipelines. For example, in the biosynthesis of the desmamides (Fig. 7), a recently discovered group of lipoglycopeptides, a FAAL domain misannotated as an A domain by antiSMASH^102^ is proposed to load a hexanoic acid started unit onto an ACP, as part of a PKS module. This is followed by chain elongation using malonyl-CoA and, through the activity of a ketoreductase, the generation of a linear eight-carbon β-hydroxy acyl-ACP intermediate. Another C2 unit is condensed to obtain a 10-carbon unit, followed by a reduction of its β-keto group and methylation by the methyltransferase DsmH. In the biosynthetic hypothesis, DsmI and DsmJ as NRPS introduce the corresponding amino acids and the cyclized metabolite is finally released through activity of a thioesterase. Surprisingly, no ACP could be identified between the FAAL domain and the first PKS module.^40^

### 3.5 Biochemical characterization of FAAL domains

Several biochemical methods have been developed for the analysis of adenylating enzyme activities that can be translated to FAAL activity, which is useful for determining substrate scope, binding and kinetics, to understand their reaction mechanism or to determine substrate preference in competition assays. Such methods include *in vitro* assays followed by chromatographic separations for product detection and liquid chromatography-mass spectrometry (LC-MS) analysis (*e.g.* CylA in cylindrocyclophanes biosynthesis^45^), indirect measuring through release or exchange of pyrophosphates (PPi) (*e.g.* FbnG in fabrubactin biosynthesis^74^), radiolabelling followed by TLC analysis in the study of mycobacterial FAAL domains^12^ or loading of acyl carrier proteins with the corresponding fatty acid substrate (*e.g.* NocH in nocuolin A biosynthesis^99^).

#### 3.5.1 Phosphate release assays

Since FAALs and other adenylating enzymes catalyze reactions where an AMP moiety is transferred from ATP to a substrate, PPi is released and can then be hydrolyzed to produce inorganic phosphate (Pi). Using phosphate release kits, the release of Pi correlates with the activity of the adenylating enzyme.^103,104^ The detection of Pi can be realized through various methods, but most release kits use a colorimetric method where Pi forms a complex with a reagent and absorbance is measured. In the malachite green assay, Pi reacts with molybdate under acidic conditions to form a phosphomolybdate complex which reacts with malachite green dye to form a green-coloured complex which increases absorption at 630 nm. Pi release assays are highly sensitive and can detect low levels of Pi.^105,106^ For example, in the study of the mycobacterial FadD32, the released PPi is hydrolyzed by a pyrophosphatase coupled reaction, followed by incubation with malachite green. The method enables a very high throughput which is why a large library of different enzyme inhibitors could be screened in the described work.^107^

#### 3.5.2 Radiolabelling of substrates

Another method to measure FAAL activity is through the formation of radiolabelled ACP-substrate species. The assay contains an activated ACP-protein and radiolabelled substrates (^3^H or ^14^C for fatty acids). Activated holo-ACP is obtained through incubation with the promiscuous Sfp (a phosphopantetheine transferase) which transfers a phosphopantetheine (Ppant) onto the conserved serine residue in the ACP. The adenylate is then covalently attached to the thiol group of the Ppant arm forming a thioester. After incorporation of the substrate, non-reacted substrate and other components can be separated from the radiolabelled acyl-ACP product that can then be detected and quantified, reflecting the activity of the FAAL enzyme.^6^ In the analysis of mycobacterial FadD32, the FAAL involved in mycolic acids biosynthesis, radiolabelled fatty acids were incubated with the enzyme, followed by separation of reaction products by TLC and detection by autoradiography.^81^

#### 3.5.3 Mass spectrometry-based methods

Several LC-MS based methods have been developed and optimized over the years to analyze ACPs and their loaded substrates. One method is the direct LC-MS analysis of intact ACPs which involves injection of intact ACP protein into a LC-MS system followed by analysis of the mass difference between the non-substrate-loaded apo-ACP and the loaded holo-ACP.^108^ For example, in the characterization of FAAL enzyme involved in the biosynthesis of cylindrocyclophanes, direct analysis of the intact ACP was carried out to determine the loading capacity of the corresponding FAAL, CylA.^45^ Standards were prepared for the apo-, holo- and decanoic acid-loaded ACP and compared to the assay sample *via* HPLC separation. An alternative is bottom-up proteomics *via* Ppant-ejection.^109^ This method includes fragmentation of the ACP within the mass spectrometer and analysis of the substrate-bound ACP-fragment. In bottom-up LC-MS techniques, ACPs are digested either enzymatically with trypsin or chymotrypsin or chemically with for example cyanogen bromide. Digestion is followed by analysis in the LC-MS and fragmentation *via* MS/MS of the loaded ACP-fragment and search for the phosphopantetheine-containing fragment.

## 4 Deorphanization of FAAL-containing BGCs and future applications

### 4.1 Diversity and discovery of FAAL-containing orphan BGCs

Bacterial lipopeptides are a group of secondary metabolites with special interest due to their antibacterial, antifungal, anticancer and antiviral bioactivities.^110^ The diversity of biological activities can be explained by their amphipathic molecular structure allowing micellar interaction with cell membranes of microorganisms.^111^ The first lipopeptide discovered and confirmed to have a FAAL involved in its biosynthesis was mycosubtilin produced by *Bacillus subtilis*.^89^ Other well-studied examples of cyclic lipopeptides with FAAL-mediated lipoinitiation of their biosynthesis are iturin, bacillomycin and puwainaphycin (Table 2). Due to their prevalence in cyanobacteria, FAAL function and FAAL-containing BGCs in these organisms have received some attention. Galica *et al.*^51^ searched publicly available genomes for gene clusters that encode FAALs and investigated the distribution of those gene clusters among cyanobacteria, considering phylogeny, ecology, and habitat. In their study, they found more than half (56%) of the lipopeptide BGCs analyzed to be present in heterocyst-forming cyanobacteria (Nostocales). In contrast, only 10% of Synechococcales strains showed positive for these BGCs. Galica *et al.*^51^ also observed an occurrence of lipopeptide BGCs in around 16% of publicly available genomes from cyanobacteria while it was reported that NRPS and NRPS/PKS gene clusters would have an abundance of around 51–56% in their genomes.^112,113^ Genome size correlated with the number of NRPS-containing gene clusters underlining the interest into cyanobacterial genomes of larger size. The same study showed the abundance of lipopeptide and herewith FAAL-containing BGCs especially in substrate-associated cyanobacteria with higher diversity in their domain organization than those coming from planktonic cyanobacteria.^51^

Several software programs for annotation of BGCs in genome data are currently available, for example antiSMASH,^102^ PRISM^114^ and ClusterFinder^4^ to name a few. Among these, antiSMASH is the most widely used bioinformatics tool designed to identify and analyze BGCs in bacterial and fungal genomes.^102^ It annotates biosynthetic signature amino acid sequences such as the “CAL” domain which in most cases corresponds to a FAAL-domain. While genome mining approaches to discover FAAL-containing BGCs are currently available, uncovering the associated natural product is seldom straightforward.^51^ In many cases, the BGCs are not expressed under typical laboratory conditions, but also accurate structural predictions from the BGC composition and architecture are necessary to match it against an orphan natural product or even to discover it in, for example, metabolomics data.^115^

In summary, FAAL-containing orphan BGCs reflect an untapped reservoir of natural products diversity with significant potential for the discovery of novel bioactive compounds, such as lipopeptides, which may be accelerated by improving structural predictions and through a better knowledge of BGC regulation in the producing organisms.

### 4.2 Substrate specificity and prediction

A better understanding of FAAL specificities would improve predicting the final natural product structures and therefore their discovery. This would also be of special interest since fatty acid chain length influences the properties such as the cytotoxicity of natural products. For example, Saurav *et al.*^116^ analyzed the effect of the fatty acid chain length on the cytotoxicity of puwainaphycins. It was observed that the lipophilicity of the fatty acid residue is essential for the compound's cytotoxicity and thus for the interaction with the plasma membrane.^117^

Initial attempts to determine substrate specificities of the acyl-adenylate superfamily were based on active site residues profiles.^118^ Using a computational approach, a total of 15 amino acid positions were defined as specificity-determining residues for each enzyme class. The sequential order of these 15 residues was defined as the active site profile in the training set and by using sequence alignments and specific scoring matrices, substrate preference was assigned based on the highest scoring. The authors were able to predict substrate preference for different subfamilies of proteins. For FAALs, these predictions included preference for medium (*n* = 2–10) and long chain (*n* = 12–16) fatty acid incorporation.^118^

Machine learning tools have been used to predict the function and substrate specificity of ANL enzymes more accurately.^2^ These tools include the prediction of substrates for NRPS adenylation (A) domains which has unlocked the possibility of accessing the biosynthetic potential of uncultured microorganisms by connecting a predicted natural product with its BGC.^119–121^ For example, bioinformatic predictions have led to the discovery of the BGC for leinamycin family compounds^122^ and helped to predict the structure of several lipopeptides based on the A-domain sequence.^123^ In another study, researchers developed an ensemble of substrate-specific Hidden Markov Models (HMMs) to classify and predict specificity of A-domains in NRPSs and acyltransferase domains in PKSs. Models were trained based on known domain sequences with annotated substrate specificities. The HMM approach showed superior performance compared to traditional methods. Predictions were evaluated against experimentally characterized datasets and demonstrated strong reliability.^124^ Novel attempts of bioinformatics guided substrate prediction were based on latent semantic indexing (LSI) techniques that are used in text mining. These analyzed and predicted protein properties based on sequence data only by identifying patterns and correlations that could successfully predict the substrate specificity of A-domains with high accuracy.^125^ Another bioinformatics approach for substrate prediction of NPRS domains was based on virtual screening of A-domains. Authors employed evaluation and ranking of potential substrates based on their binding affinity to the A-domains, created a library of potential substrates and used docking simulations to analyze for the fit of each substrate into the binding sites of different A-domains. This virtual screening approach predicted substrate preference with high accuracy and was matching experimental data.^126^

Existing tools still have limitations, such as the inability of predicting with confidence the chain length of lipid tails incorporated into lipopeptides by specifically FAAL enzymes. To some extent, this might be explained by the fact that FAALs have shown to have broad substrate acceptance. For example, the FAAL encoded on the corramycin BGC was predicted to load fatty acids ranging from pentanoic acid to dodecanoic acid. *In vitro* results confirmed the activation of numerous linear fatty acids with various lengths. Still, highest activity was observed for decanoic acid and decreased activity for hexanoic acid underlining that a substrate preference of FAALs does exist.^95^ The finding that lipopeptides often occur with different fatty acid chain length substitutions can be explained by the broad substrate tolerance of FAALs in general. Compared to FAALs, FACLs also exhibit substrate acceptance for different fatty acyl chain lengths, but they tend to have a more restricted substrate range due to their role in providing specific fatty acyl-CoA intermediates to primary metabolism.^118^ A more recent prediction algorithm for adenylating enzymes, AdenylPred, was developed by Robinson *et al.*^2^ This computational prediction algorithm was designed to predict the substrate specificity and function of class I adenylate-forming enzymes by using machine learning techniques (random forest approach). AdenylPred aids to advance the discovery of natural products by analyzing adenylation domains amino acid sequences and predicting the enzymes' role. Within-class accuracy was highest for FAALs and NRPS adenylation domains due to the larger amount of experimental data for those protein families. Still, the algorithm could only predict fatty acid substrate ranges rather than a precise fatty acid chain length.^2^

Since no β-oxidation pathway has been yet identified in cyanobacteria, a method for the discovery of fatty acid harbouring natural products that allows for direct labelling of the cyanobacterial metabolome followed by dereplication has recently been developed. This method is based on the incorporation of stable isotope-labelled fatty acids into the cyanobacterial lipidome and thus enabling the deorphanization of yet unknown BGCs. Using this novel strategy, analogues of hapalosin and the new family of compounds, nocuolactylates, were uncovered.^28^

The biotechnological potential of FAAL enzymes was recently demonstrated in the context of daptomycin biosynthesis.^127^ Researchers swapped the original FAAL enzyme with non-native homologs, specifically CylA and HmqF, which improved fatty acyl specificity and resulted in higher daptomycin purity (70% and 90% compared to 40%), despite the FAALs originating from phylogenetically distant bacteria. Additionally, the authors fused FAALs to ACPs, leading to remarkable increases in daptomycin titers. This highlights the potential of FAAL enzymes to enhance production titers and to aid in the design of novel natural products.

Our current understanding of FAAL substrate specificity is still rudimentary. For example, the development of prediction algorithms based on machine learning could be enhanced by expanding the dataset of characterized FAALs. Additionally, the generation of comprehensive substrate libraries and conducting mutagenesis experiments would provide more precise specificity profiles.

Synthetic biology offers opportunities to design customized FAAL variants through directed evolution and modular assembly of biosynthetic pathways. Integrating FAAL into non-natural NRPS or PKS systems could enable the synthesis of novel and more bioactive products. However, challenges such as accurately mimicking native interactions between FAALs and their downstream ACPs as well as the limited understanding of post-translational modifications remain.

## 5 Conclusions

First identified in *Mycobacterium tuberculosis*, the discovery of FAALs marked a significant advancement in understanding lipid metabolism in bacteria. Over time, FAALs have been found across all three domains of life – bacteria, archaea, and eukaryotes – highlighting their essential role in lipid metabolism. A breakthrough in differentiating FAALs from the structurally similar FACLs was the identification of the FAAL specific insertion (FSI), a unique amino acid sequence found exclusively in FAALs.

The resolution of several FAAL crystal structures has further illuminated their reaction mechanisms and substrate-binding properties. FAALs are now known to participate in the biosynthesis of a variety of natural products, particularly within Cyanobacteria, Actinobacteria, Pseudomonadota, Firmicutes, and Myxococcota. As genome mining continues, it is expected that more FAAL-associated natural products will be discovered.

FAALs demonstrate functional versatility by existing either as stand-alone, didomain proteins or as components of multienzyme complexes. The availability of diverse methods to characterize FAAL proteins has accelerated the discovery and deorphanization of related BGCs. Additionally, the ability to predict FAAL substrates enhances the connection between BGCs and their corresponding natural products, further driving research into natural products.

In summary, FAALs, once overlooked, have now emerged as widely distributed enzymes that play a critical role in the biosynthesis of various natural products, particularly polyketides and lipopeptides. Their unique function in channelling fatty acids into secondary metabolism makes them a promising target for metabolic engineering and enzyme modification to create novel fatty-acylated natural products in bacteria.

## 6 Data availability

The structural alignments were performed using UCSF ChimeraX (version 1.3). All structural data used in this study was retrieved from the Protein Data Bank (PDB). No new experimental data was generated for this review.

## 7 Author contributions

A. L. and P. N. L. conceptualized and wrote the article.

## 8 Conflicts of interest

There are no conflicts to declare.
